# Prime-Boost Strategies in Mucosal Immunization Affect Local IgA Production and the Type of Th Response

**DOI:** 10.3389/fimmu.2013.00128

**Published:** 2013-05-29

**Authors:** Fabio Fiorino, Elena Pettini, Gianni Pozzi, Donata Medaglini, Annalisa Ciabattini

**Affiliations:** ^1^Laboratorio di Microbiologia Molecolare e Biotecnologia, Dipartimento di Biotecnologie Mediche, Università di Siena, Siena, Italy

**Keywords:** prime-boost, T cell priming, mucosal immune response, nasal immunization, subcutaneous immunization, cytokines

## Abstract

Combinations of different delivery routes for priming and boosting represent vaccination strategies that can modulate magnitude, quality, and localization of the immune response. A murine model was used to study T cell clonal expansion following intranasal (IN) or subcutaneous (SC) priming, and secondary immune responses after boosting by either homologous or heterologous routes. T cell primary activation was studied by using the adoptive transfer model of ovalbumin-specific transgenic CD4^+^ T cells. Both IN and SC immunization efficiently elicited, in the respective draining lymph nodes, primary clonal expansion of antigen-specific CD4^+^ T cells that disseminated toward distal lymph nodes (mesenteric and iliac) and the spleen. After boosting, a significant serum IgG response was induced in all groups independent of the combination of immunization routes used, while significant levels of local IgA were detected only in mice boosted by the IN route. Mucosal priming drove a stronger Th1 polarization than the systemic route, as shown by serum IgG subclass analysis. IFN-gamma production was observed in splenocytes of all groups, while prime-boost vaccine combinations that included the mucosal route, yielded higher levels of IL-17. Memory lymphocytes were identified in both spleen and draining lymph nodes in all immunized mice, with the highest number of IL-2 producing cells detected in mice primed and boosted by the nasal route. This work shows the critical role of immunization routes in modulating quality and localization of immune responses in prime-boost vaccine strategies.

## Introduction

Most licensed vaccines include a priming dose and at least one boost of the same immunogen to generate an effective immune response in terms of increasing magnitude, quality, and localization. The prime-boost concept, traditionally based on the same vaccine given multiple times over a certain period (homologous prime-boost), is currently applied also to the administration of the vaccine antigen in different formulations (heterologous prime-boost) (Lu, 2009; Radosevic et al., 2009). Initially developed to combine recombinant DNA priming with viral vector boosting (McShane, 2002; Ranasinghe and Ramshaw, 2009), the heterologous prime-boost approach has now been applied to many different combinations of delivery systems and has been tested in several clinical trials (Hill et al., 2010; Paris et al., 2010; Rowland and McShane, 2011; O’Hara et al., 2012; Sheehy et al., 2012). Results from the AIDS vaccine trial RV144 performed in Thailand show that a prime-boost regimen of two genetically engineered vaccine candidates, that had failed on their own, lowered the rate of HIV infection by about 31% (Rerks-Ngarm et al., 2009). Prime-boost approaches are applied also in the field of vaccination against tuberculosis, in which many human studies are investigating the utility of a “booster” subunit vaccine, comprising both protein-adjuvant combinations or recombinant viral vectors, to be used in BCG vaccinated people (Rowland and McShane, 2011; Rowland et al., 2013; Tameris et al., 2013). The heterologous prime-boost approach can also be achieved by combining different routes of vaccination. This strategy, principally based on the combination of mucosal and parenteral delivery, has the advantage of inducing immune responses in both the local and systemic compartments that are as strong or stronger than those resulting from homologous mucosal or parenteral vaccination alone (McCluskie et al., 2002; Glynn et al., 2005; Mapletoft et al., 2010; Pattani et al., 2012). Indeed, the use of homologous or heterologous routes can impact on the efficiency and the localization of the immune response to a vaccine formulation.

Targeting mucosal sites by vaccination is important considering that over 90% of infections occur at or through mucosal surfaces. Mucosal immunization has been demonstrated to efficiently elicit humoral and cellular responses both locally and systemically, as observed in animal models and in humans (Lycke, 2012). The nasal route of immunization has largely proved to be effective in inducing memory immune responses both systemically and locally, i.e., in the respiratory, genital, and intestinal tracts (Zuercher, 2003; Neutra and Kozlowski, 2006; Ciabattini et al., 2010). Limitations to the use of the nasal route are generally related to the choice of adequate mucosal adjuvants and delivery systems whose potency needs to be carefully balanced with their potential toxicity (Couch, 2004; Lewis et al., 2009).

For the design of a prime-boost vaccination strategy, it is important to characterize the early events that happen during the primary immune response, such as the CD4^+^ T cell clonal expansion upon antigen-MHC class II complex recognition. T cell priming influences both B and T cell activation and the subsequent differentiation into effector and memory cells. Indeed, the frequency of primed antigen-specific CD4^+^ T cells has been shown to correlate with the intensity of the secondary responses and may be a predictor of vaccine immunogenicity in humans (Galli et al., 2009). We have previously investigated the antigen-specific CD4^+^ and CD8^+^ T cell primary activation upon mucosal immunization with soluble antigen mixed with mucosal adjuvants or delivered by recombinant bacteria, using the adoptive transfer model system (Medaglini et al., 2006; Ciabattini et al., 2008b, 2010, 2011; Pettini et al., 2009). Intranasal (IN) immunization, with both recombinant bacteria or soluble antigen with CpG adjuvant, elicited an early antigen-specific clonal expansion of T cells within lymphoid tissue draining the immunization site and the subsequent dissemination of these activated cells (CD45RB^−^CD44^+^CD62L^+^) toward distal lymph nodes through the blood (Ciabattini et al., 2008b, 2010, 2011). Homing of nasally primed T cells in distal iliac lymph nodes was CD62L-dependent, while entry into mesenteric lymph nodes depended on both CD62L and α4β7, as shown by *in vivo* antibody-mediated inhibition of T cell trafficking (Ciabattini et al., 2011).

In the present work, the T cell clonal expansion following IN or subcutaneous (SC) priming and the secondary immune response after boosting by either the homologous or heterologous routes was studied in mice. Ovalbumin (OVA) mixed with the synthetic TLR9 agonist CpG oligodeoxynucleotide (ODN) 1826, widely described as an effective adjuvant for both parenteral and mucosal immunization (Tengvall et al., 2006; Bode et al., 2011), was used as the model vaccine formulation. The antigen-specific CD4^+^ T cell priming after IN or SC immunization was studied employing the adoptive transfer model of OVA-specific transgenic CD4^+^ T cells (Kearney et al., 1994; Pettini et al., 2009). The local and systemic antibody responses, as well as the T cell response in the spleen and in draining lymph nodes, were characterized following homologous or heterologous combination of prime-boost routes.

## Materials and Methods

### Animals

Eight-weeks old female OT-II TCR-transgenic (H-2^b^), C57BL/6J, and BALB/c mice, purchased from Charles River (Lecco, Italy), were maintained under specific pathogen-free conditions in the animal facilities at the University of Siena, and treated according to national guidelines (Decreto Legislativo January 27, 1992 n. 116, implementing 86/609/CEE Directive). All animal studies were approved by the Ethics Committee “Comitato Etico Locale dell’Azienda Ospedaliera Universitaria Senese” and the Italian Ministry of Health (number 4/2011, July 20, 2011).

### Adoptive transfer of transgenic CD4^+^ T cells and priming studies

Adoptive transfer experiments were performed as previously described (Pettini et al., 2009). Briefly, lymphocytes collected from OT-II transgenic mice were enriched for CD4^+^ T cells and stained with carboxy-fluorescein diacetate succinimidyl ester (CFSE, 7.5 μM, Invitrogen). An amount of 2.5 × 10^6^ of CFSE-labeled T cells was injected into the tail vein of each recipient mouse. After 24 h, C57BL/6J mice were immunized with OVA grade V (Sigma-Aldrich; 25 μg/mouse) and CpG ODN1826 (TCC ATG ACG TTC CTG ACG TT, hereafter CpG ODN; Eurofins MWG Operon, Ebersberg, Germany; 20 μg/mouse) by the IN or SC routes. IN immunized mice were lightly anesthetized by intraperitoneal injection of tiletamine and zolazepam hydrochloride (Zoletil 20, Laboratoires Virbac, France, 6 mg/kg) and xylazine (Xilor 2%, Bio 98 Srl, Italy, 3 mg/kg), held in a vertical position and then inoculated with drops into a single nostril with a total volume of 20 μl of Phosphate Buffered Saline (PBS) solution. SC immunization was performed dorsally in the region of the neck in a total volume of 100 μl of PBS.

Groups of three mice were sacrificed 0, 3, 5, and 7 days following immunization. Cervical (CLN), mediastinal (MedLN), axillary (AxLN), iliac (ILN), mesenteric (MLN) lymph nodes, and spleen (SPL) were harvested from each mouse and individually mashed onto a nylon screen. Cells were washed twice in Hanks’ Balanced Salt Solution (HBSS, Gibco), incubated with Fc-blocking solution [0.5 mg CD16/CD32 mAb (clone 93) (eBioscience, USA), 5% v/v mouse serum, 5% v/v rat serum, 0.2% w/v sodium azide (all from Sigma-Aldrich) in 100 ml of HBSS] for 30 min at 4 °C, and stained with PerCP-conjugated anti-mouse CD4 (clone RM 4–5, BD Pharmingen) for 30 min at 4 °C. Samples were analyzed by flow cytometry (FACScalibur, Becton Dickinson, San Diego, CA, USA). Data analysis was performed by using Flow Jo software (Tree Star, Ashland, OR, USA).

### Immunization using prime-boost combinations and sample collection

BALB/c mice (six per group) were primed by the IN or SC route with OVA grade V (100 μg/mouse) mixed with the adjuvant CpG ODN (20 μg/mouse) at days 0 and 10 and boosted 7 weeks later by the homologous (IN/IN, group 1 and SC/SC, group 3) or by the heterologous route (IN/SC, group 2 and SC/IN, group 4), while group 5 was left *naive* as control. Immunizations were performed in a volume of 20 or 100 μl of PBS for IN and SC immunizations, respectively. Blood samples were taken from the temporal plexus on days 0 and on weeks 3, 5, 7, 9, 11, 13, and sera were stored at 4 °C. Nasal washes were performed at week 13, as previously described (Ciabattini et al., 2008a).

### ELISA

Serum OVA-specific IgG, IgG1, and IgG2a were determined by ELISA, as previously described (Medaglini et al., 2001), coating plates with OVA (4 μg/ml). OVA-specific IgG titers were expressed as the reciprocal of the highest dilution with an optical density value ≥0.2 after background subtraction. The concentration of OVA-specific IgG1 and IgG2a was calculated using a standard curve of mouse myeloma IgG1 and IgG2a (Southern Biotechnology Associates, Birmingham, AL, USA).

Ovalbumin-specific IgA were assessed in nasal washes coating plates with OVA (4 μg/ml) and diluting samples 1:2 in duplicate. Alkaline-phosphatase-conjugate goat anti-mouse IgA (Southern Biotechnology Associates) were added diluted 1:1000, and the optical density was recorded using Thermo Scientific Multiskan FC Microplate Photometer. The concentrations of OVA-specific IgA were calculated against a standard curve of mouse myeloma IgA (Southern Biotechnology Associates).

### IL-2 ELISPOT

Lymphocytes were collected from cervical and mediastinal lymph nodes and spleen at week 13. For IL-2 ELISPOT, 96-well nitrocellulose plates (Milititer HA, Millipore, USA) were coated overnight at 4 °C with 5 μg/ml of purified rat IL-2 monoclonal antibody (BD Pharmingen, USA). Free binding sites were blocked with complete RPMI. Pooled lymphocytes were seeded in triplicate starting from 3 × 10^6^ cells/well and diluted up to 3 × 10^5^ cells/well. Cells were stimulated with 90 μg/ml of OVA for 18 h at 37 °C in 5% CO_2_. After washing, plates were incubated with biotinylated anti-mouse IL-2 monoclonal antibody and then with streptavidin-horseradish peroxidase (all from BD Pharmingen, USA). The enzyme reaction was developed by adding 1 mg/ml of the substrate 3,3′-diaminobenzidine (Sigma-Aldrich). Spots were counted with an EliScan immunospot analyzer (A.EL.VIS, Hannover, Germany), and data were expressed as spot forming units (SFU) per million of cells.

### Cytokine assay

IFN-γ, IL-4, and IL-17A production were assessed in culture supernatants of restimulated splenocytes by Bio-Plex cytokine immunoassay. Pooled splenocytes from each group were cultured with 90 μg/ml of OVA in complete RPMI for 72 h at 37 °C in 5% CO_2_, and supernatants were collected and stored at −80 °C. IFN-γ, IL-4, and IL-17A cytokines were detected using the 3-plex assay system (Bio-Rad), following the manufacturer’s protocol, and analyzed by Luminex 100 (Bio-Rad). Cytokine concentrations were calculated based on standard curve data using Bio-Plex Manager software (version 4.0).

### Statistical analysis

Percentages of antigen-specific proliferating T cells after IN or SC priming were compared using the two-tailed Student’s *t*-test. Sera were tested individually and values were expressed as mean ± standard error of the mean (SEM). Statistical differences between antibodies among groups were assessed using one-way analysis of variance (ANOVA) and Tukey’s post test for multiple comparisons on log-transformed data. The two-tailed Student’s *t*-test was used for analyzing differences in IgG titers at different time points and for comparing values of OVA-specific IgG1 and IgG2a between nasally and subcutaneously primed mice. IL-2 production among different groups was analyzed using one-way ANOVA and Tukey’s post test on log-transformed data. Statistical significance was defined as *P* ≤ 0.05. Graphpad 4.0 software was used for analysis.

## Results

### Antigen-specific primary activation of CD4^+^ T cells

**Figure 1:**
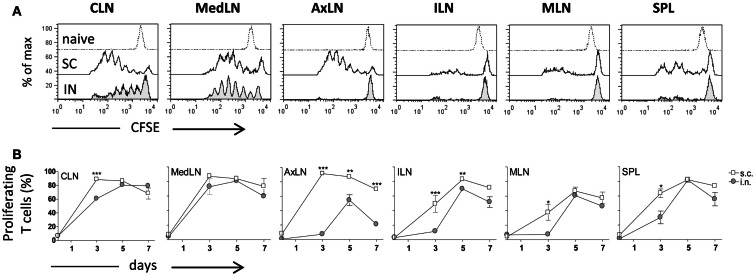
**Clonal expansion of transgenic CD4^+^ T cells after IN or SC priming**. C57BL/6J mice, adoptively transferred with CFSE-labeled OT-II CD4^+^ T cells, were primed by the IN or SC routes with OVA mixed with the adjuvant CpG ODN. OVA-specific proliferation was analyzed in T cells collected from cervical (CLN), mediastinal (MedLN), axillary (AxLN), iliac (ILN), mesenteric (MLN) lymph nodes, and spleen (SPL) at different time points. **(A)** OVA-specific proliferation of OT-II CD4^+^ T cells detected by CFSE dilution in lymphoid organs 3 days following IN (bottom histogram) or SC (middle histogram) priming or in untreated mice (top histogram). **(B)** Time-course analysis of the clonal expansion of OT-II CD4^+^ T cells following IN (filled circle) or SC (empty square) priming with OVA and CpG ODN. The clonal expansion was analyzed on days 0, 3, 5, and 7 post-immunization. Values reported on the *y* axis indicate the percentage of proliferating OT-II cells compared to the total transgenic cells detected in each lymph node or spleen. Values are expressed as means ± SEM of 2 independent experiments, each performed with three animals. Two-tailed Student’s *t*-test was used for comparing values of proliferating T cells between nasal and subcutaneous primed mice at each time point. *P ≤ 0.05, **P ≤ 0.01, and ***P ≤ 0.001.

Both IN and SC routes of immunization yielded an efficient primary activation of CD4^+^ T cells as demonstrated using the adoptive transfer model of CFSE-labeled OVA-specific transgenic CD4^+^ T cells. Three days after both IN and SC priming with OVA and CpG ODN, antigen-specific clonally expanded T cells were observed in CLN and MedLN (Figure 1A). The T cell proliferation in draining CLN was more rapid following SC than IN immunization, as shown by the higher number of cells within advanced cell generations (Figure 1A) and the higher percentage of proliferating T cells at day 3 (*P*  ≤ 0.001; Figure 1B). Priming by the SC, but not the IN route, induced T cell expansion in AxLN, that act as draining lymph nodes upon immunization in the dorsal neck region (Figures 1A,B). A significantly higher percentage of proliferating cells after SC priming was also observed in distal lymph nodes and in the SPL (*P*  ≤ 0.001 for ILN; *P*  ≤ 0.05 for MLN and SPL versus IN immunized mice; Figure 1B). The presence of primed T cells at distal sites is due to migration of proliferated T cells from draining lymph nodes, as previously demonstrated (Ciabattini et al., 2011). Nevertheless, studying the clonal expansion of OVA-specific T cells at days 5 and 7 after immunization, we observed similar percentages of dividing cells between the two groups (Figure 1B). The percentage of dividing cells peaked at day 5 following both IN and SC priming (with about 80% of divided T cells in CLN, MedLN, ILN, and SPL), and persisted for at least 7 days (Figure 1B). As expected, a significant difference was maintained only in AxLN (*P*  ≤ 0.01; Figure 1B).

### Secondary immune responses following homologous or heterologous prime-boost combinations

#### Systemic antibody response

**Figure 2:**
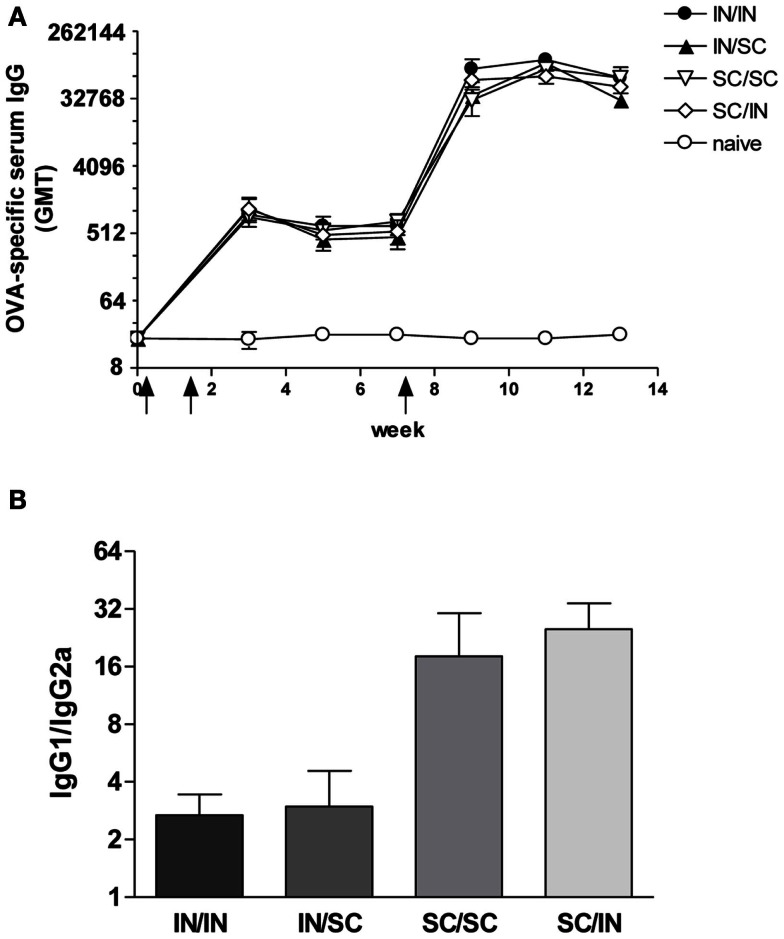
**OVA-specific serum antibody response**. BALB/c mice were immunized with OVA mixed with the adjuvant CpG ODN at days 0 and 10 by the IN or SC route followed by a boost at week 7 by the homologous or heterologous route. Antibody response was assessed by ELISA on individual serum samples. **(A)** OVA-specific IgG response assessed in samples collected at weeks 0, 3, 5, 7, 9, 11, and 13. Antibody titers were expressed as the reciprocal of the highest dilution with an OD value ≥0.2 after background subtraction. Values are reported as GMT ± SEM. **(B)** Ratio of OVA-specific IgG1 and IgG2a subclasses assessed in final serum of each animal. Data are reported as mean ± SEM for each group.

Ovalbumin-specific serum antibody responses were assessed in mice primed with OVA and CpG ODN by the IN or SC route and boosted at week 7 by the homologous (IN/IN and SC/SC) or heterologous (IN/SC and SC/IN) routes. A significant antigen-specific IgG response, with a geometric mean titer (GMT) of about 1000, was observed in all groups after priming compared to *naive* mice (*P*  < 0.001; Figure 2A). Boosting resulted in a rapid increase of the IgG titers which was comparable in all groups of immunized animals. Serum antibody levels persisted in all groups 6 weeks after boosting (Figure 2A). Even if the immune responses detected in mice primed-boosted by the IN route were higher than the other groups, no statistically significant difference was observed. To study the type of Th response, the distribution of IgG subclasses was assessed in the final sera (week 13) of all animals. Mice primed by the SC route (SC/SC and SC/IN) produced higher levels of OVA-specific IgG1 antibodies compared to mice primed by the nasal route (IN/IN and IN/SC) which in turn developed a significantly higher response of OVA-specific IgG2a (*P* ≤ 0.05; Table 1), as shown by the ratio of IgG1/IgG2a subclasses (Figure 2B). This isotype switching suggests a stronger Th1 polarization in mucosally primed mice.

**Table 1:** **Serum total and OVA-specific IgG subclasses in immunized animals^a^**.

Animal group^b^	IgG1 (μg/ml)		IgG2a (μg/ml)	
	
	
	OVA-specific	Total	OVA-specific	Total
IN/IN	363 ± 121	2780 ± 493	147 ± 26^*c^	850 ± 86
IN/SC	266 ± 132	2740 ± 322	148 ± 43^*^	909 ± 216
SC/SC	435 ± 68	3304 ± 130	71 ± 25	715 ± 54
SC/IN	643 ± 141	2805 ± 121	65 ± 29	596 ± 24
Untreated	<0.011	790 ± 96	<0.03	192 ± 27

^a^Total and OVA-specific IgG1 and IgG2a antibodies assessed in the final sera (week 13) of each animal. Data reported as mean concentration (μg/ml) ± SEM of each group.^b^Animals were primed and boosted by homologous (IN/IN and SC/SC) or heterologous (IN/SC and SC/IN) routes; IN, intranasal immunization; SC, subcutaneous immunization.^c^Two-tailed Student’s *t*-test was used for comparing values of OVA-specific IgG1 and IgG2a between nasally *versus* subcutaneously primed mice. ^*^P ≤ 0.05.

#### Local antibody response

**Figure 3:**
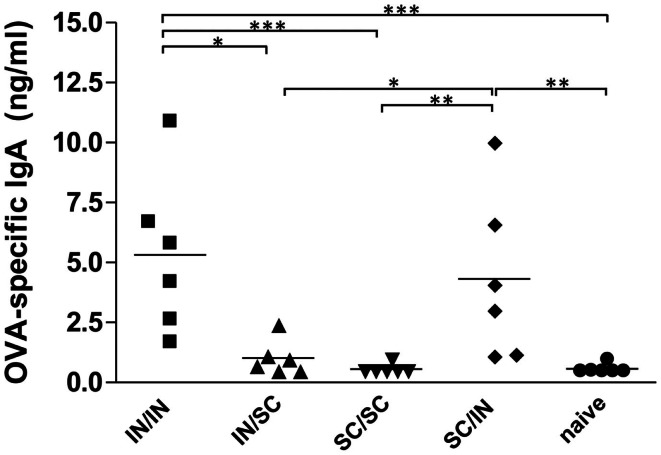
**OVA-specific local IgA**. BALB/c mice were immunized with OVA mixed with the adjuvant CpG ODN at days 0 and 10 by the IN or SC route followed by a boost at week 7 by the homologous or heterologous route. OVA-specific IgA were assessed in nasal washes collected at sacrifice (week 13). Bars represents the mean value of OVA-specific IgA concentration per each group. One-way ANOVA and Tukey’s post test for multiple comparisons were used for comparing antibody response in different groups. ^*^P < 0.05, ^**^P < 0.01, and^***^P < 0.001.

The study of antigen-specific IgA in nasal washes after IN or SC boosting revealed that local antibody responses were essentially dependent on mucosal boosting. A significantly higher IgA response was observed in mice boosted by the nasal route, with values of 5.32 ng/ml (IN/IN) and 4.32 ng/ml (SC/IN) of OVA-specific IgA, compared to the *naive* group (*P* < 0.001 and *P* < 0.01, respectively) and not in mice boosted by the SC route (SC/SC and IN/SC; Figure 3). Moreover, the local IgA response elicited in mice boosted by the nasal route was statistically higher than the one induced in mice boosted by the systemic route (Figure 3).

#### Cellular immune response in spleen and lymph nodes

**Figure 4:**
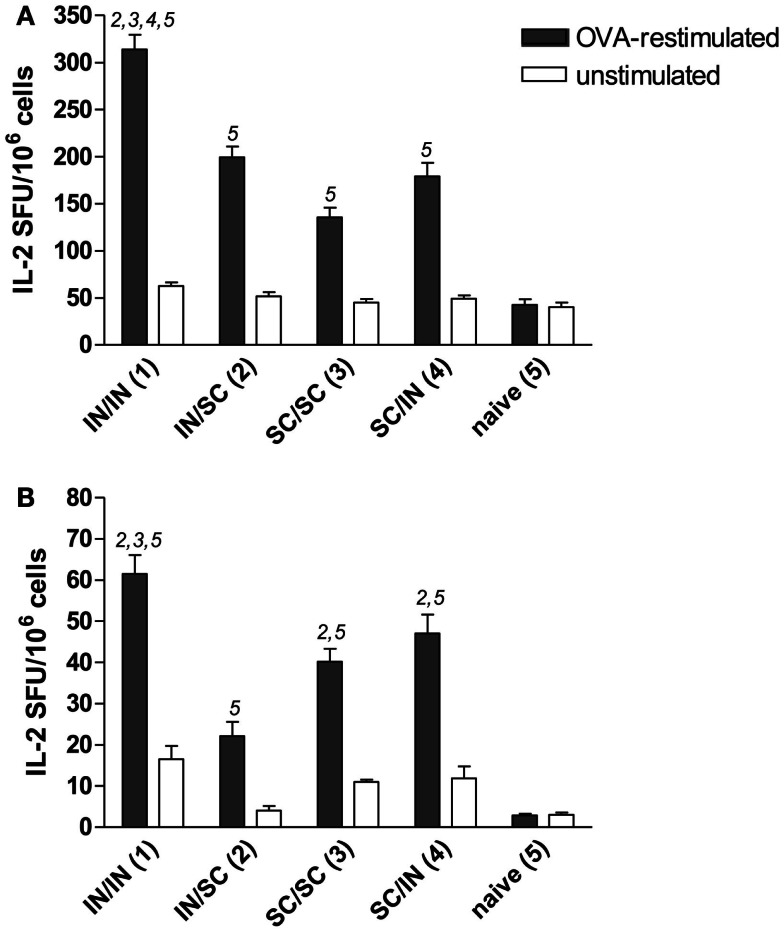
**OVA-specific lymphocyte response determined by IL-2 ELISPOT assay**. BALB/c mice were immunized with OVA mixed with the adjuvant CpG ODN at days 0 and 10 by the IN or SC route followed by a boost at week 7 by the homologous or heterologous route. Lymphocytes were obtained from spleens **(A)** and pooled cervical and mediastinal lymph nodes **(B)** at week 13, and the IL-2 production was evaluated following OVA restimulation by ELISPOT assay. The number of antigen-specific spot forming units (SFU) of OVA-restimulated (black histograms) and unstimulated (white histograms) cells is shown. Bars represent the mean number of SFU/10^6^ cells ± SEM of triplicate samples. One-way ANOVA and Tukey’s post test for multiple comparisons was performed for all groups. The numbers above the error bars indicate which groups (1–5) are statistically different. Statistical significance was defined as *P* < 0.05.

Antigen-specific memory cells were detected in the spleen and draining lymph nodes (cervical and mediastinal) of mice immunized by all different prime-boost combinations. After *in vitro* restimulation with OVA antigen, all groups developed a significantly higher number of IL-2 producing cells compared to *naive* mice (Figures 4A,B). Mucosal priming and boosting generated the highest number of responder lymphocytes in both spleen (315 SFU/10^6^ cells; *P* < 0.05 *versus* all the groups; Figure 4A) and draining lymph nodes (61 SFU/10^6^ cells; *P* < 0.05 *versus* mice boosted by the SC route; Figure 4B). In the absence of antigenic restimulation, no group developed a significant IL-2-response (Figures 4A,B).

**Figure 5:**
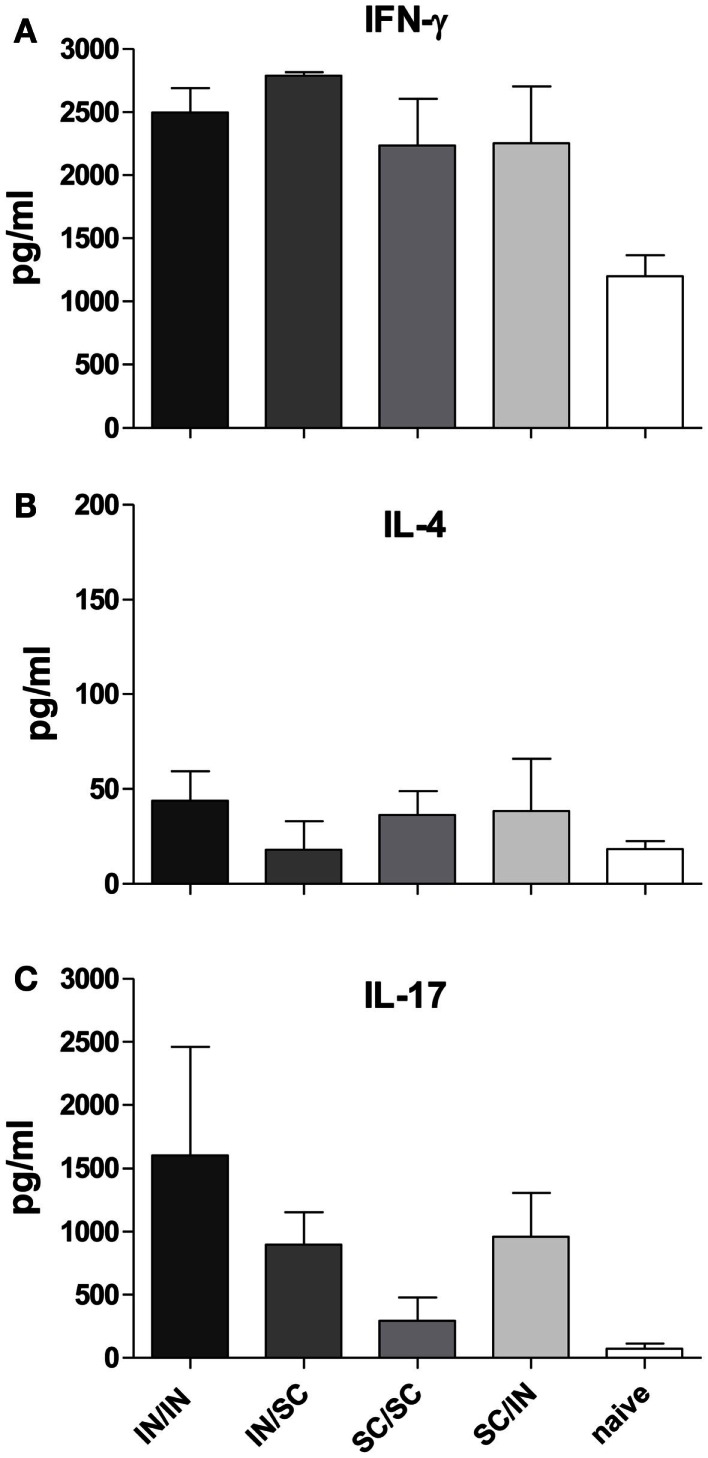
**Cytokine production in culture supernatant of splenocytes**. IFN-γ **(A)**, IL-4 **(B)**, and IL-17A **(C)** were detected by Bio-Plex immunoassay in culture supernatants of pooled splenocytes restimulated with OVA (as described in Materials and Methods). Bars represent the mean concentration ± SD of duplicate samples.

In order to characterize the quality of the cellular response, the production of IFN-γ, IL-4, and IL-17A cytokines was assessed in the culture supernatant of splenocytes pulsed with OVA. An increase of IFN-γ levels was observed in all prime-boost combinations compared to the control group (Figure 5A), while low levels of IL-4 production were detected (Figure 5B). Interestingly, higher levels of IL-17A were induced by immunization schemes including the mucosal route compared to the homologous SC/SC combination (Figure 5C).

## Discussion

In the present work we show that prime-boost combinations by IN and SC routes affect the local and systemic immune responses in mice.

Essential parameters of long-term vaccine efficacy are the persistence of antibodies and the generation of immune memory cells. T cells are crucial to the induction of high-affinity antibodies and immune memory, thus being important effector players since the primary immunization. Here, we have explored the primary expansion of antigen-specific CD4^+^ T cells following IN and SC immunization in mice adoptively transferred with OT-II transgenic cells. A more rapid proliferation within draining lymph nodes and dissemination toward distal sites was observed in subcutaneously primed mice. However, 7 days after immunization the percentage of antigen-specific divided cells, detected in draining and distal lymphoid compartments, was not significantly different between the two routes, except for AxLN, that act as draining lymph nodes following SC but not IN immunization. Subcutaneously injected soluble antigens are rapidly carried from the injection site into draining lymph nodes, through afferent lymphatic vessels, where they are taken up by resident dendritic cells that produce antigen-MHC II complexes, with a peak reached 3 h after immunization (Itano et al., 2003). Therefore, it is possible that the antigen-presentation upon SC immunization is faster than the IN delivery route. The proliferative response observed here in C57BL/6J mice adoptively transferred with transgenic OT-II cells is comparable to the one observed in BALB/c mice adoptively transferred with DO11.10 transgenic cells following nasal immunization with either soluble OVA and CpG ODN (data not shown) or OVA-expressing recombinant *S. gordonii* (Medaglini et al., 2006).

The use of the adoptive transfer technique for studying *in vivo* T cell primary activation allows to enrich the very low frequency of *naïve* antigen-specific T cells, and therefore to characterize *in vivo* their clonal expansion in different organs. Obviously, the transfer of a high number of *naive* T cells into recipient mice may alter the physiological conditions; to overcome this possible limitation and assess the antigen-specific primary T cell activation *in vivo* following vaccination, we are currently further investigating mucosal T cell priming with MHC class II tetramers specific for relevant vaccine antigens.

Comparable levels of serum IgG antibodies were induced by both IN and SC primary immunization, which persisted for at least 7 weeks after priming. Booster vaccination induced a significant and rapid increase of the serum IgG antibody levels independently of prime-boost combinations. Interestingly, mucosal prime-boost schedule (IN/IN) was as immunogenic as the parental one (SC/SC). Regarding the IgG subclasses switching, it is known that Th1/Th2 polarization is influenced by factors such as the genetic predisposition of mice, the vaccine formulation (McCluskie et al., 2002) and the immunization route. BALB/c mice are genetically polarized toward a Th2 response (Finnegan et al., 1999), while the CpG adjuvant promotes the generation of a Th1 response (Chu et al., 1997; Tengvall et al., 2006). In this study, by maintaining the same vaccine formulation for priming and booster immunizations, we analyzed the role of the immunization route, observing that (i) IN and SC routes differently affect the immunoglobulin class switching, and (ii) the isotype switching is driven by the priming and not booster immunization. Mice primed by the nasal route developed higher OVA-specific IgG2a antibodies compared to parenterally primed mice that in turns produced higher IgG1. Nasal priming induced therefore a stronger Th1 polarization, as already observed with other antigens and vaccine formulations (Ricci et al., 2000; Medaglini et al., 2001). The Th1 polarization was confirmed by the high production of IFN-γ and very low release of IL-4 in culture supernatants of splenocytes in all immunized groups. A third type of inducible T cell immunity involves IL-17 production, a cytokine initially found to play a central role in inflammation and autoimmunity and now recognized as an important cytokine involved in normal responses to pathogens, such as those of the respiratory tract (Lu et al., 2008; Sonnenberg and Weiner, 2009), and in protective immunity induced by vaccination (Bettelli et al., 2007; Moffitt et al., 2011). Here we report the production of IL-17A in mice immunized by the IN route, while a lower IL-17A response was observed in mice primed-boosted by the SC route. These data, showing that nasal immunization promotes IL-17 production, are in line with previous studies performed with different adjuvants including CpG (Zygmunt et al., 2009; Bielinska et al., 2010; Arias et al., 2012).

The analysis of the prime-boost combinations showed also that the booster route, more than the priming route, affects the induction of local immune response, supporting previous data obtained with different vaccine formulations (McCluskie et al., 2002). Local antigen-specific IgA, indicative of effector plasma cells in the nasal mucosa, were detected only when mice were boosted by the nasal route. Mucosal priming enhanced the local IgA response when nasally boosted, but was not sufficient without the mucosal booster immunization as shown by the IN/SC group. Interestingly, mice primed and boosted by the SC route did not produce significant nasal IgA antibodies, even if an efficient T cell priming had occurred in cervical and mediastinal lymph nodes, as demonstrated by the T cell clonal expansion data. This suggests that the nasal boost is essential for stimulating plasma cells capable of homing to the nasal mucosa and releasing local IgA. Lymphocytes collected from cervical and mediastinal lymph nodes were responsive to *in vitro* restimulation with OVA in all groups, with the highest number of IL-2 producing cells detected in mice boosted by the nasal route. These data are in line with what was recently observed by the vaginal route, where vaginal booster immunization was necessary to elicit an effector response in the vaginal tract, both in terms of local antibodies and T cells (Marks et al., 2011). Thus, our data confirm the importance of the booster immunization route to elicit a mucosal effector response.

In summary, in our system we observed that (i) both IN and SC immunizations prime antigen-specific CD4^+^ T cells and that these cells can be efficiently boosted by either homologous or heterologous routes; (ii) the route used for priming and not for boosting influences the Th1 or Th2 skewing, with a stronger Th1 polarization in mucosally primed mice; (iii) mice primed and/or boosted by the nasal route produce higher levels of IL-17A than mice primed-boosted systemically; and (iv) systemic immune responses are efficiently elicited by all prime-boost combinations, while local effector responses are mainly dependent on mucosal boosting.

Taken together, these results highlight the critical role of the priming and booster immunization routes in modulating the quality and the localization of the secondary immune responses, and that prime-boost routes should be considered for the rational design of a vaccination strategy.

## Conflict of Interest Statement

The authors declare that the research was conducted in the absence of any commercial or financial relationships that could be construed as a potential conflict of interest.
